# Green Extraction and Preliminary Biological Activity of Hydrolyzed Collagen Peptides (HCPs) Obtained from Whole Undersized Unwanted Catches (*Mugil cephalus* L.)

**DOI:** 10.3390/molecules28227637

**Published:** 2023-11-17

**Authors:** Valentina Orlandi, Lorenzo Dondero, Federica Turrini, Giulia De Negri Atanasio, Federica Grasso, Elena Grasselli, Raffaella Boggia

**Affiliations:** 1 Department of Pharmacy, University of Genova, Viale Cembrano 4, 16148 Genova, Italy; valentina.orlandi@edu.unige.it (V.O.); federica.grasso@edu.unige.it (F.G.); 2 Department of Earth, Environmental and Life Sciences, University of Genova, Corso Europa, 26, 16132 Genova, Italy; lorenzo.dondero@edu.unige.it (L.D.); giulia.denegriatanasio@edu.unige.it (G.D.N.A.); elena.grasselli@unige.it (E.G.); 3 National Center for the Development of New Technologies in Agriculture (Agritech), 80121 Napoli, Italy; 4 Interuniversity Center for the Promotion of 3R Principles in Teaching and Research (Centro 3R), 56122 Pisa, Italy; 5 National Biodiversity Future Center (NBFC), 90133 Palermo, Italy

**Keywords:** fish side-streams, hydrolyzed collagen peptides, green extraction, ultrasound, enzymes, biological activity

## Abstract

Considering the global increase in fish consumption, the growing side-streams coming from the fish supply chain (e.g., skin, fins, tail, heads…), also including undersized or “unwanted catches”, have been recently proposed as source of high-value bioactive compounds (e.g., peptides and fatty acids). In this case study, hydrolyzed collagen peptides (HCPs) were extracted from different parts of *Mugil cephalus* L. using environmentally friendly techniques such as ultrasounds and enzymatic treatments. Both a mixed biomass derived from the skin, fins, and tail, and a whole fish, were considered as starting biomass, simulating the unsorted processing side-streams and an undersized/unwanted catch, respectively. The extracted HCPs were purified in fractions (<3 KDa and >3 KDa) whose yields (about 5% and 0.04–0.3%, respectively) demonstrated the efficiency of the hydrolysis process. The extraction protocol proposed allowed us to also isolate the intermediate products, namely the lipids (about 8–10%) and the non-collagenous proteins (NCs, 16–23%), whose exploitation could be considered. Each sample was characterized using Sircol, UltraViolet-Spectra, and hydroxyproline assay, and the viability of their collagen fractions was tested on human endothelial cells. Significant effects were obtained at a fraction of <3 KDa, in particular at a concentration of 0.13 µg/mL. The T-scratch test was also performed, with positive results in all fractions tested.

## 1. Introduction

In recent years, the exponential growth of the world’s population has led to a massive increase in aquatic food production, both by capture fishery and aquaculture. According to the Food and Agriculture Organization’s (FAO) reports, their production reached a record of 214 million tons in 2020, including 178 million tons of aquatic animals and 36 million tons of algae [1].

As a result of rising fish consumption, the consequent production of food waste is also increasing. The FAO has estimated that up to 35% of global fish production is lost or wasted every year, representing a huge environmental issue with a significant economic impact [2]. It has been estimated that only 30–50% of the fish weight is utilized [3], while the fins, heads, skin, tails, bones, and viscera are discarded, further exacerbating the problem of overfishing [4].

The fish supply chain produces increasing quantities of side-streams, which are represented by the waste of the final consumer, the by-products with low economic value such as scraps from industrial processing, and the so-called “unwanted catches”. Unwanted catches are undersized fish or fish with other characteristics that make them not suitable for sale such as a low economic value, damaged fish or those belonging to a protected species, and for these reasons they are often released into the sea, causing economic and environmental problems [5].

The urgency for change in production systems towards more eco-compatible alternatives has led the EU Commission to elaborate and to approve the so-called “Blue Growth” [6], a long-term planning strategy for the sustainable growth of the marine and maritime sectors. This “blue transformation” also has, among its main targets, the upgrading of the aquatic food chain throughout, reducing loss and waste to ensure the achievement of the UN Sustainable Development Goals of the Agenda 2030.

Since the most common residues of the fish supply chain contain on average 40–60% proteins, 20–30% ashes and 7–20% lipids (dry weight) [7], the valorization of these side-streams, in view of a “zero waste” economy, has attracted the attention of many researchers, aiming to recover as much as possible in terms of high-value bioactive compounds, such as peptides, fatty acids and other bioactive metabolites [8], that can be potentially exploited in different fields including food, pharmaceutical, cosmetic and biomaterials [2].

Recently, the global demand for collagen-derived products has been increasing due to the growing interest in the potential health benefits of collagen and hydrolyzed collagen peptides (HCPs) [7,9,10,11,12,13,14].

The main sources for the extraction of collagen and HCPs are still represented by bovine and porcine animals (especially from their skins, bones and cartilage). However, during the last few years, the use of collagen derived from these animal discards is raising concern due to infectious diseases, such as bovine spongiform encephalopathy (BSE) and foot-and-mouth disease (FMD), and religious constraints [9,12]. Marine collagen represents a valid alternative thanks to its safety, easy extractability, low production costs, scalability of the process, water solubility and aforementioned bioactive properties.

HCPs are obtained from native collagen through an acid/alkali or enzymatic hydrolysis process, that generally involves proteolytic enzymes. The process of hydrolyzing collagen molecules results in the production of compounds that exhibit distinct biological activities and physicochemical properties. HCPs exhibit a different isoelectric point, viscosity, and film-forming capability, as well as higher digestibility and bioavailability, and lower allergenicity compared to native collagen [15]. Thus, HCPs possess unique properties due to their low molecular weight, typically in the range of 3–6 KDa or even lower than 3 KDa. These biological activities include anti-aging, anti-photoaging, moisturizing, the promotion of wound healing, anti-hypertensive, antioxidant, and antimicrobial/antimycotic activities [16]. Considering the valorization of co-products, the utilization of HCPs presents an excellent opportunity for application in both the pharmaceutical and cosmetic industries, offering versatile and promising compounds [17,18].

The aim of this study was to demonstrate the possibility of exploiting unwanted catches, as an example of undifferentiated biomass, for the extraction of bioactive compounds (i.e., collagen, HCPs) that can be used in various sectors such as cosmetics. In fact, it is important to underline that very often unwanted catches are not subjected to the filleting procedure, and the separation of their different parts could represent a process too expensive to be sustainable.

In this research, for the first time, the extraction of marine HCPs starting from different biomasses of *Mugil cephalus* L. was proposed using environmentally friendly techniques such as ultrasounds (UAE, Ultrasound-Assisted Extraction) and enzymatic treatments (EAE, Enzymatic-Assisted Extraction). Particularly, two different samples, namely (1) whole undersized fish, representing a specimen with little commercial value (under-sized unwanted catches, F1), and (2) skins, fins and tails, as an example of usual residual biomass resulting from the filleting process (F2), were investigated after a preliminary freeze-drying step, that stabilizes these highly perishable biomasses and, at the same time, could offer advantages also from a logistical point of view. These two types of samples have been also compared, in terms of proximate analysis, with the edible portion of *Mugil cephalus* L., namely the fillet.

A flow-chart was proposed, both suggesting the possibility to exploit the collateral products obtained during the process (i.e., lipids and non-collagenous proteins) and exploring the possibility to start from unsorted biomass (i.e., whole undersized unwanted catches) instead of starting from the usual separate discarded side-streams (i.e., skin, scales, tails, …). It is important to underline that very often, unwanted catches are not subjected to the filleting procedure, and the separation of their different parts could represent a process too expensive to be sustainable; therefore, the exploitation of this undifferentiated biomass could have a high added value.

The so-obtained peptides (HCPs) were been tested to estimate their biocompatibility and proliferative promoting activity on an HECV endothelial cell line over a period of 24 h to investigate them as promising components for the wound healing activity by T-scratch assay. This study is part of the preliminary data used as proof of concept to elaborate a larger project called “EcoeFISHent”, a European Union’s Horizon 2020 project (Innovation Action, Grant agreement ID: 101036428) concerning the valorization of fish side-streams for the development of cosmetic and nutraceutical products, starting from previously dehydrated biomass [19].

## 2. Results and Discussion

### 2.1. Preliminary Characterization of Samples

The proximate analysis was carried out on the freeze-dried samples (F1, F2) and compared with the edible portion, namely the fillet. The results of the residual moisture, crude proteins, lipids, and ashes, expressed as g/100 g of freeze-dried samples, are reported below in Table 1.

The moisture contents of all samples after freeze-drying were about 4–5%, compatible with the technological treatment carried out in order to stabilize these high-perishable biomasses. The lowest crude protein content was found in the sample made up of skin, fins, and tails (F2); instead, the crude protein content of the whole fishes (F1) was, as expected, on average between F2 and the edible portion (fillet). Analogously, the ashes content was highest (about 21 g/100 g) in the usually discarded parts (skin, fins, and tails, F2) and the lowest value (about 11 g/100 g) was registered in the edible portion (fillet). As expected, the value of the whole fish (F1) was again in the range between the results of F2 and the edible portion (fillet). Even if these values are for guidance only, since they referred to a small sampling of mullets (5 kg) and they could be influenced from many factors (i.e., fish size, fish age, fish habitat, kind of capture…), all the investigated samples can be considered as a good source for protein/peptide extraction.

In the literature, the proximate composition reported by Suvanich et al. [20] concerning side-streams of another fish supply chain was made up of 58% of proteins, 22% of ashes, and 19% of lipids. As far as the protein content is concerned, the results reported in the literature agreed with the results obtained in this study, justifying the further exploitation of F1 and F2 samples in terms of potential peptide recovery. Particularly, the possibility to start from unsorted biomass (F1), also under-sized whole fishes as an example of unwanted catches, represents important advantages in terms of sustainability and costs.

Concerning the lipidic content, the extraction yields obtained from the defatting with ethanol, as a green pre-treatment step for HCP extraction, are reported in Table 1.

Moreover, the lipid content of the samples was also monitored by applying the procedure described by Smedes et al. [21,22], with us obtaining results comparable to those obtained from the abovementioned defatting step (11–12 g/100 g). It should be considered that both defatting with ethanol and using the method proposed by Smedes et al. [21] may underestimate the lipid content of samples. However, these methods have made it possible to avoid the use of toxic halogenated solvents and save energy compared to the common Folch/Bligh&Dyer methods [23] or the official Soxhlet one [24].

### 2.2. Pre-Treatment and Recovery of the Isolated Intermediate Fractions

Generally, the collagen extraction procedure starting from fishery biomass, as reported by Ali et al. [25] and by Jafari et al. [26], includes the following steps: washing and cutting the raw material, chemical pretreatment, extraction, precipitation, and freeze-drying of the extracted collagenic fraction.

In this study, the protocol proposed by Ali et al. [25], was adapted, with some significant modifications due to the dehydrated starting materials instead of fresh or frozen starting biomass, and due to the aim of the recovery of as much as possible of the isolated intermediate fractions (fish oil and non-collagenous proteins (NCPs)). Usually, from this type of biomass, the lipidic fraction is removed using 10% butyl alcohol after the preliminary non-collagenous protein’s isolation, performed using 0.1 M NaOH [18,25,27,28,29].

Conversely, in the proposed protocol, the defatting step was moved as the first step of the pretreatment, and was performed using a green solvent (ethanol 96%) at room temperature to potentially exploit the fish oil extracted, limiting its hydrolysis and oxidation as much as possible, which would be worsened by contact with aqueous solution (favoring saponification and oxidation reactions).

**Figure 1:**
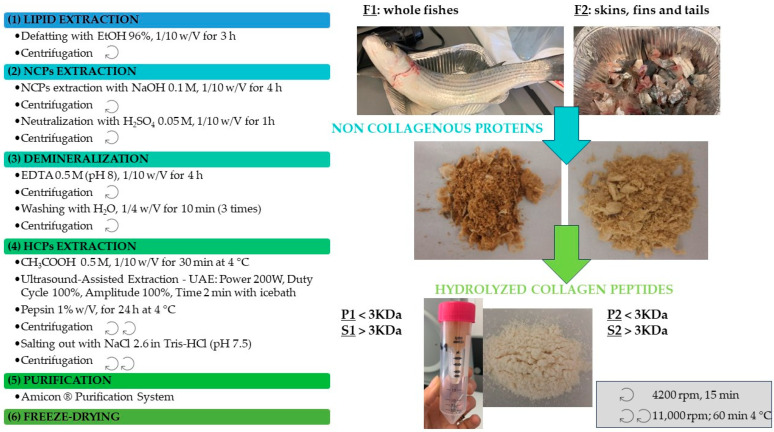
Flow-chart of the sample pre-treatment, extraction, purification, and formulation of HCPs.

The flow-chart of the whole process is reported in Figure 1.

Furthermore, to obtain the complete exploitation of the extraction intermediates, the freeze-drying of the defatted non-collagenous proteins (NCPs) isolated after the removal of the lipid fraction was proposed. The choice of using NaOH during the pretreatment is supported by the review of Jafari et al., 2020 [26], that underlines the higher swelling ability of sodium hydroxide that facilitates the extraction of collagen by increasing the transfer rate of the mass in the tissue matrix. The yields of NCPs extracted from F1, F2 and from the edible portion (fillet) are summarized in Table 2. Data were expressed both as NCPs/total freeze-dried sample weight and as NCPs/total freeze-dried protein weight.

As expected, fillets (F3) represent the major source of NCPs; nevertheless, the recovery of this fraction starting both from F2 and F1 provide comparable results, whose values can justify their useful exploitation and recovery.

The third pretreatment step was the demineralization, performed using an aqueous solution of the chelating agent of the divalent cation ethylenediaminetetraacetic acid (EDTA). As reported from Jafari et al., 2020 [26] the demineralization of this raw material (starting biomass) is required to enhance the collagen extraction efficiency, especially from the parts characterized by high amount of mineral material (i.e., bones, cartilage, and scales).

### 2.3. Ultrasound-Assisted Extraction of Hydrolyzed Collagen Peptides (HCPs)

The pellets obtained after the pretreatment steps were suspended in acetic acid 0.5 M at 4 °C for collagen extraction (step 4, Figure 1). In order to increase the extraction yields, an ultrasound treatment was applied, as suggested by Ali et al. [30], Hong et al. [31], and Skaik et al. [32], since cavitation may allow the solvent to penetrate through the biomass more effectively. The mechanical impact, caused by the collapse of the cavitation bubbles, guarantees more effective penetration of the solvent into the matrix due to the increase in the contact surface; moreover, other mechanisms such as fragmentation, erosion, capillarity, detexturation, and sonoporation are responsible for the enhancement of yield [33]. However, the sonication time was limited to a few minutes to limit energy consumption, also in view of a potential future scale-up of the process.

Furthermore, after the sonication process, the enzyme pepsin was added to the suspension to additionally increase the extraction yield. Pepsin cleaves the telopeptide regions of the triple helix, increasing the leaching of collagen peptides in the solution and the yield of the extraction [26].

The so-extracted HCPs were precipitated by salting out, and peptides with a molecular weight < 3 KDa were isolated by those > 3 KDa using an Amicon^®^ Pro Purification membrane system, since, according to numerous articles published in the literature, this range of molecular weights shows the most interesting bioactivities of collagen peptides, the best bioavailability, and the lowest potential allergenicity [15]. The acid concentration, enzyme concentration, solid/liquid ratios and time of extraction have been displayed in the previous figure, where the flow-chart of the whole process is schematized.

After final freeze-drying (step 6 of Figure 1), the following peptide extracts (HCPs) were obtained: P1 and P2 < 3 KDa, and S1 and S2 > 3 KDa named, correspondingly, according to the starting biomass F1 and F2, respectively.

The ponderal yields of the HCP extracts, both <3 KDa (P1 and P2) and >3 KDa (S1 and S2), expressed both as HCPs/total freeze-dried sample weight and as HCPs/total freeze-dried protein weight, were reported in Table 3.

Concerning the HCPs < 3 KDa (P1 and P2), the results show that the higher yield of extraction, expressed as the HCPs with respect to the total protein content (dry weight), was obtained starting from F2 sample (P2), containing skin, fins and tails, which represents the common discarded parts usually exploited as starting material for the collagen recovery [28,34,35]. P1, obtained originally from whole fish, gave similar results to P2, suggesting that the whole fish, as an example of unwanted catches, even when not previously sorted, could be a promising source of HCPs, such as the parts usually separated in filleting processes (F2). These results demonstrate the potential use of fish side-streams even if not previously separated, an operation that requires an enormous effort in terms of time and money, especially for unwanted catches. Even if the yields of extraction could change enormously depending on the fish and on the part used as the starting material, as reported in the review of Coppola et al. [36], the obtained results (in terms of ponderal yields of about 4–8%) can be considered as satisfactory respect to the data reported in the literature for *Mugil cephalus* L. In fact, the extraction yield of acid soluble collagen (ASC) from *Mugil cephalus* L. reported in the literature was about 0.40% [36,37]. On the other hand, concerning the yields of extractions of HCPs > 3 KDa (S1 and S2), they were lower than the corresponding fractions of HCPs < 3 KDa, confirming that the hydrolysis process was quite effective.

### 2.4. Sircol and Hydroxyproline Tests

The total amount of collagen was detected using the Sircol collagen assay (SCA). As reported in Table 4, the extracts with a molecular weight < 3 KDa (P1 and P2) presented a concentration of collagen which was homogeneous and in accordance with that of the commercial collagen used as the control (CTRL+), while the fraction with a molecular weight > 3 KDa (S1 and S2) reported a higher amount of collagen. Indeed, the HCP fractions with higher molecular weights (>3 KDa, S1 and S2) presented much more collagen due to the incomplete hydrolyzation of the extracts. The SCA is based on the amino acid binding property of Sirius red (SR F3B; CI 35782), an anionic dye with sulphonated acid side-chain groups that reacts with the side-chain groups of basic amino acids [38,39]. SR is used widely as a selective histochemical stain for collagen in normal and pathological tissue [38,40,41,42,43]., It is well known that the SCA can overestimate the concentration of collagen [44]. For this reason, the total amount of hydroxyproline was also evaluated, showing that the S1 and S2 fraction present a higher concentration of Hyp compared to the P1 and P2, which confirmed the results obtained from the SCA technique but with a lower concentration. The fraction > 3 KDa presented the amount of Hyp in the same range of the commercial collagen tested (CTRL+). Nevertheless, it is possible to notice that from all the fractions studied, it is possible to obtain collagen. It is important to highlight that the commercial sample of marine collagen peptides provided by a cosmetic industry and used as the standard (CTRL+) had a molecular weight < 3 KDa. Moreover, the marine origin and the type of collagen were not declared on its technical sheet.

### 2.5. Spectrophotometric Characterization of HCPs Extracts

#### 2.5.1. UltraViolet (UV) Spectra

**Figure 2:**
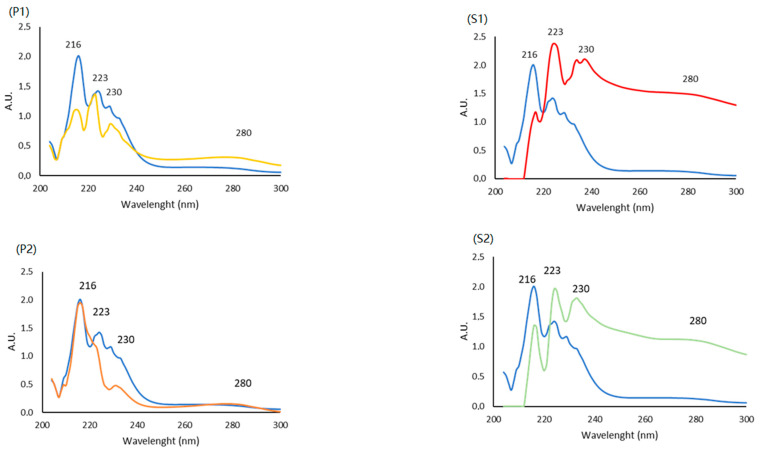
UV spectra of P1, P2, S1 and S2 vs. a commercial sample < 3 KDa collagen peptides of a non-declared marine origin as the positive control (CTRL + blue line, P1 yellow line, P2 orange line, S1 red line and S2 green line).

UV spectra represent a simple way to characterize collagen; usually, a negative absorbance at 204 nm and a prominent peak at 230 nm due to the triple helix are reported [45]. The UV spectra (from 200 to 300 nm) of each fraction at the same concentration (1 mg/mL), both <3 KDa (P1, P2) and >3 KDa (S1, S2), are reported in Figure 2. Every sample was compared to a <3 KDa commercial collagen used as the reference (CTRL+, in blue line).

All the fractions present peaks in the region 210–230 nm associated with the groups C=O, -COOH and CONH_2_ [46,47], typical of proteins due to their polypeptide chains, and a hump at 280 nm resulting from the presence of a small amount of aromatic residues (such as tyrosine, phenylalanine and tryptophan) [48,49], which could be due to a presence of non-collagenous proteins [50]. Despite the spectra similarity, in terms of peak, the different absorbance level suggests small structural and/or compositional differences among the fractions obtained. In our case, the maximum peak at 230 nm is not high, precisely because the molecular weight was <3 KDa (P1 and P2). Indeed, the fraction > 3 KDa present that peculiar trend (S1 and S2).

#### 2.5.2. Fourier-Transform Spectroscopy (FTIR)

**Figure 3:**
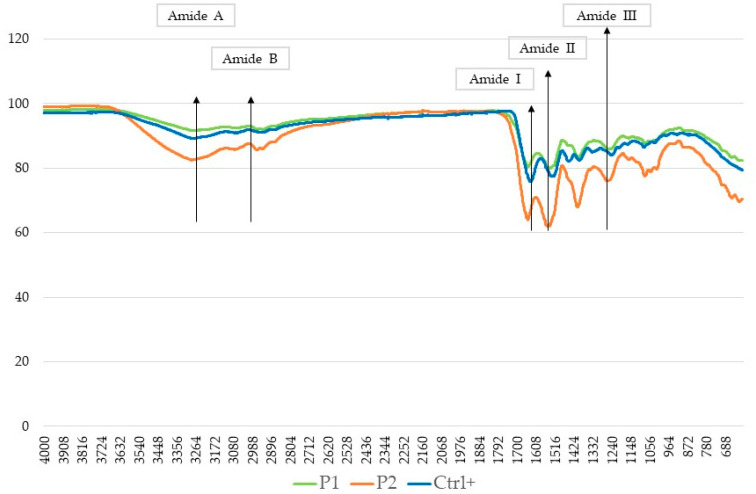
FTIR spectra of P1 and P2 vs. a commercial sample < 3 KDa collagen peptides of a non-declared marine origin as the positive control (CTRL+, blue line).

**Figure 4:**
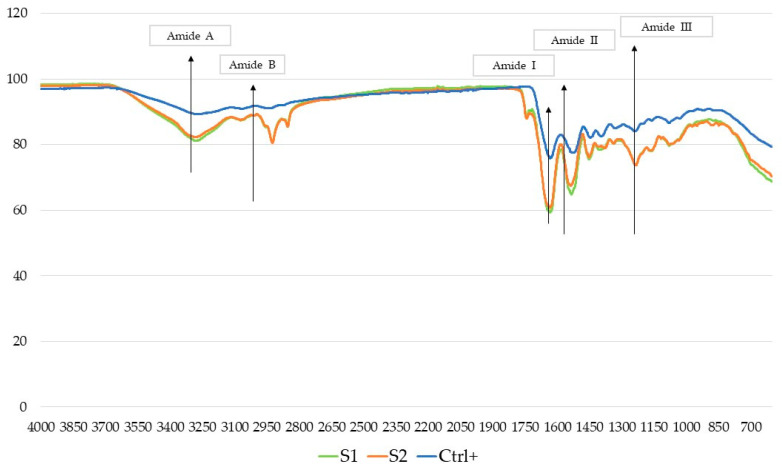
FTIR spectra of S1 and S2 vs. a commercial sample > 3 KDa collagen peptides of a non-declared marine origin as the positive control (CTRL+, blue line).

FTIR spectra of the HCPs extracts < 3 KDa, namely P1 and P2, and HCPs extract > 3 KDa (S1 and S2), are shown in Figure 3 and Figure 4, respectively.

The FTIR analysis is based on the natural vibration frequencies of chemical bonds present in molecules [51]. The amides A, B, I, II and III are the characteristic peaks of typical collagen peptide structures, indicative of the amino acid composition in the collagen molecule [52]. The amide A band indicates the stretching vibrations of the free N-H group and the presence of hydrogen bonds, and, according to the literature, was found in the range of 3300–3400 cm^−1^ [53]. The amide B band (approximately 2900 cm^−1^) indicates the asymmetrical stretching of both the CH_2_ and –NH3+ groups [54]. The amide I, with a wavelength value in the range of 1600–1700 cm^−1^, is associated with a carbonyl group stretching vibration coupled to a carboxyl group. The amide II (1500–1600 cm^−1^) represents the N-H bending vibration and the C-N stretching vibration. Finally, the amide III band value (1200–1300 cm^−1^) is associated with the C-N stretching vibration and N-H bending vibration, which are involved in the complex intermolecular interactions of collagen [55].

The absorbances of all the amide bands are higher in the fractions > 3 KDa compared to the standard (CTRL+) and to the fractions < 3 KDa, indicating that the enzymatic hydrolysis produces changes in their configuration. The amide A bands are much more evident in fraction > 3 KDa than in the control (CTRL+), and in fractions < 3 KDa. Also, amide B bands are evident in the samples > 3 KDa (S1 and S2); on the other hand, they are less evident in the sample P1 < 3 KDa and in the control (CTRL+). Amide I bands are present in all the spectra; instead, Amide III bands are less evident in all samples. In conclusion, the FTIR spectra suggest that the structures were only slightly changed in each isolated fraction.

### 2.6. Cellular Activity

#### 2.6.1. Cell Viability (MTT)

To assess the biocompatibility of the collagen fraction, the MTT assay [3-(4,5-dimethylthiazol-2-yl)-2,5-diphenyltetrazolium bromide] was used to test its cell viability on human endothelial HECV cells, and all the treatments were compared to a negative control (untreated) and a positive control (commercial collagen with a molecular weight < 3 KDa) after 24 h.

**Figure 5:**
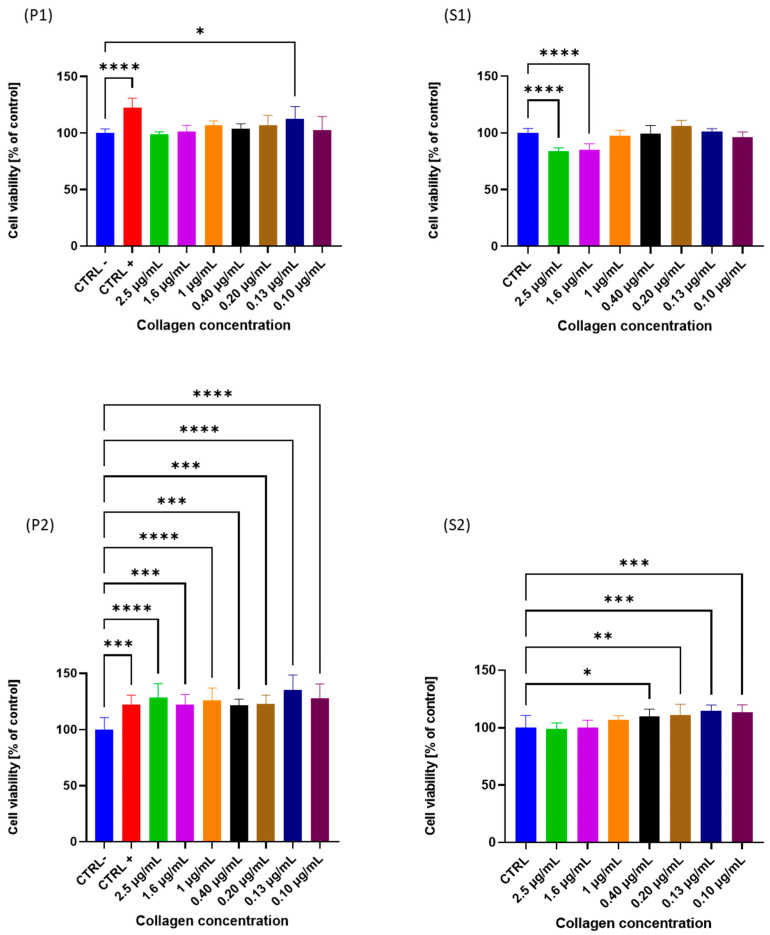
Viability of different collagen fractions (P1, S1, P2 and S2) on HECV cells after 24 h. Data are expressed as a percentage of the negative control; values mean ± SD from at least 3 independent experiments. The significant differences are shown as symbols on bars (**** *p* ≤ 0.0001, *** *p* ≤ 0.001, ** *p* ≤ 0.01, * *p* ≤ 0.05 vs. CTRL−).

As reported in Figure 5, results indicate that, after treatment with increasing concentration of P1 fraction, the viability of HECV cells was not different from the negative control except for the cells treated with a concentration of 0.13 µg_collagen_/mL. Cells treated with the P2 fraction, on the contrary, presented an increasing viability at all the concentrations tested after 24 h. Considering the results, it was possible to observe no significant difference in cells treated with all P3 fraction concentrations.

Moreover, since P2 was able to promote a proliferative effect to any of the tested concentrations, P1 stimulated cell proliferation only at 0.13 µg/mL. The cells treated with the fraction S1 presented a statistically significant decrease for the cells treated with the higher concentration (2.5 and 1.6 µg_collagen_/mL) compared with the untreated cells. Meanwhile, the cells treated with the S2 fraction did not present any negative cell viability at all the concentrations tested.

It is noteworthy that none of the extracts assayed here had a negative impact on the cell viability, considering all the concentrations tested.

#### 2.6.2. T-Scratch Test

Collagen is one of the higher components of the ECM in the dermis and has a role in the physiological interaction for pro-regenerative, migration, and differentiation activities. The presence of aminoacidic residues from collagenous peptides could represent a good additional booster for cellular proliferation and migration [56].

**Figure 6:**
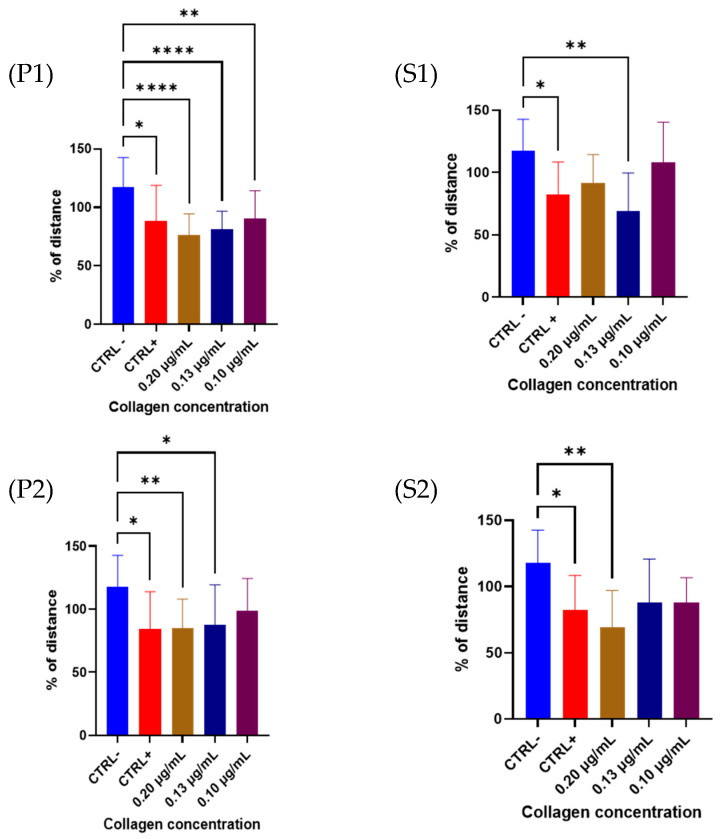
T-scratch test on HECV cells after 24 h treated with P1, P2, P1 and S2 fraction. Data are expressed as percentage of negative control; values mean ± SD from at least 3 independent experiments. The significant differences are shown as symbols on bars (**** *p* ≤ 0.0001, ** *p* ≤ 0.01, * *p* ≤ 0.05 vs. CTRL−).

T-scratch is a simple assay to evaluate the wound closure in a 2D cellular system in terms of cell proliferation and migration activities among different compounds [57]. In our case, the T-scratch assay of HECV human endothelial cells was tested in the presence of the fractions P1, S1, P2, and S2, and compared to the commercial collagen at different concentrations: 0.20, 0.13, and 0.10 µg/mL. The results suggest a significant difference between the negative control (untreated cells) and cells treated with a concentration of 0.13 µg/mL of collagen of the fractions P1, S1, and P2, while for S2, a significant difference was reported at a concentration of 0.20 µg/mL of collagen (Figure 6). Of note, cells treated with P1 presented different wound closure compared to the untreated cells at all concentrations studied. The cells in contact with the P2 fraction presented a significant difference only at concentrations of 0.20 and 0.13 µg/mL. These results suggest the synergy of different molecules, which can have an effect on cell proliferation. Indeed, in the P1 fraction (the whole fish), other compounds could be present due to the nature of the extract.

## 3. Materials and Methods

### 3.1. Samples

Fresh samples of *Mugil cephalus* L. (Mugilidae), caught in the Ligurian Sea (North-West Mediterranean, Italy), were immediately processed in order to obtain three different kinds of raw materials from which bioactive compounds were extracted: the whole fish (Fish 1: F1), including the head and viscera, which simulated the unwanted catches (or by-catches not subjected to the filleting process); the second (Fish 2: F2), consisting of skin, fins, and tails. Each sample was obtained by first roughly cutting the fish into pieces of about 1/2 cm and then grinding it in a blender (Grindomix GM200, Retsch, Haan, Germany) at 5000 rpm for 20 s until a homogeneous material was obtained. Samples were frozen at −18 °C for 48 h and then lyophilized with a freeze-dryer Buchi Lyovapor L200I S (Büchi Labortechnik AG, Flawil, Switzerland) to stabilize them over time until analyses.

### 3.2. Chemicals

Ultrapure Milli-Q water (18 MΩ) was produced by a Millipore Milli-Q system (Bedford, MA, USA) and used throughout. All chemicals and reagents were of analytical grade. Ethanol 96% and acetic acid were provided by VWR Chemicals (Radnor, PA, USA), while sodium hydroxide 0.1 M, sulfuric acid 0.05 M, EDTA (Ethylenediaminetetraacetic acid) 0.5 M, sodium chloride, pepsin (extracted from porcine gastric mucosa, EC 3.4.23.1), Hydroxiproline (PHR1939), copper sulfate, sodium hydroxide, 4-(Dimethylamino)benzaldehyde (DMAB), 2-propanol, ethanol absolute, phosphoric acid, Coomassie brilliant blue G 250, MTT (3-(4,5-dimethylthiazol-2-yl)-2,5-diphenyltetrazolium bromide), and BSA (bovine serum albumin) were supplied by Sigma-Aldrich Chemical Company (Steinheim, Germany). Dulbecco’s Modified Eagle’s Medium (DMEM, high-glucose w/l-glutamine w/sodium pyruvate), fetal bovine serum (FBS), trypsin, and Dulbecco’s Phosphate-Buffered Saline w/o calcium w/o magnesium (DPBS), used for cell culture, were purchased from Euroclone (Pero, Italy). The Sircol collagen assay kit (SCA; Biocolor Ltd., Antrim, Northern Ireland) and Hydroxiproline kit (perchlorate-free) from Cell Biolabs were used for the collagen quantification.

### 3.3. Preliminary Characterization of Samples

The residual moisture of the samples was estimated using a Sartorius thermogravimetric moisture analyzer (Marlborough, MA, USA), after comparing this analytical method with the official AOAC one (950.46 B). Ashes were determined following the AOAC official method (942.05), which consisted of a preliminary treatment in a drying-oven Binder FED53 (Goettingen, Germany) for 20 min at 105 °C, followed by ignition at 600 °C for 2 h in a Nabertherm muffle furnace LE 2/11/R7 (Lilienthal, Germany). The protein content was evaluated using the official Kjeldahl method (AOAC 981.10) using a Kjeldahl automatic distillation system K-360 BUCHI (Büchi Labortechnik AG, Flawil, Switzerland). A 6.25 conversion factor was used to calculate the protein content from the Kjeldahl nitrogen content. The lipid content was gravimetrically estimated after the defatting step, performed during the pretreatment for HCP extraction. Moreover, it was also monitored using the procedure described by Smedes et al. [21]. All measurements were performed in duplicate, and results are reported as the mean value ± standard error (Table 1).

### 3.4. Pre-Treatment

Freeze-dried samples of *Mugil cephalus* L. were processed following the experimental protocol of Moula Ali et al., 2018 [30], with some important modifications. The whole process consisted of several steps, including pre-treatment, extraction, and purification stages, which are graphically summarized in Figure 1. Lipids were removed by defatting with ethanol 96% in a solid/solvent ratio of 1/10 (*w*/*v*), with continuous stirring for 3 h at room temperature. The recovered lipid fraction was dried under vacuum (rotavapor, 37 °C) and then weighed to evaluate the extraction yield (Table 1). The ethanol used for defatting, after its recovery in the rotavapor, was recycled and reused for subsequent extractions. To remove non-collagenous proteins (NCPs), the solid residues were soaked with a solution of NaOH 0.1 M in a solid/solvent ratio of 1/10 (*w*/*v*) and stirred for 4 h at room temperature. After the alkali treatment, samples were neutralized with H_2_SO_4_ 0.05 M in a solid/solvent ratio of 1/10 (*w*/*v*), with stirring for 1 h at room temperature. The pretreated samples were further demineralized with ethylenediaminetetraacetic acid (EDTA) 0.5 M (pH 8) using a solid/solvent ratio of 1/10 (*w*/*v*) for 4 h with continuous stirring. Then, the demineralized samples were washed with cold water (1/4 *w*/*v*) and continuously stirred for 10 min. Washing was repeated three times.

### 3.5. Ultrasound-Assisted Extraction of Hydrolyzed Collagen Peptides (HCPs) 

Pretreated samples were soaked in acetic acid 0.5 M with a solid/solvent ratio of 1/10 (*w*/*v*), with continuous stirring for 30 min at 4 °C, allowing them to partially swell by holding the extraction mixture. Then, the suspension was subjected to a sonication process using an Hielscher UP200St (Teltow, Germany) equipped with a 14 mm diameter titanium probe, while using an ice bath to maintain the temperature at under 30 °C throughout the whole process. The experimental extraction conditions, which had been previously optimized by the authors on the same raw material, were summarized in Figure 1.

After the sonication treatment, to improve the extraction yield, porcine pepsin 1% (*w*/*v*) was added to the suspension and then the samples were consistently gently stirred at 4 °C for 24 h. The suspension was centrifugated at 11,000 rpm and 4 °C for 1 h using a benchtop refrigerated centrifuge model 5810R (Eppendorf, Switzerland). The collagen in the supernatant was precipitated through salting out by adding NaCl 2.6 M water solution. The pellet was dissolved in a minimum volume of acetic acid 0.5 M before undergoing the next purification process.

### 3.6. Purification, Formulation and Calculation of the HCP Ponderal Yields

All the extracts were filtered through an Amicon Stirred Cell, using a 3 KDa membrane as the exclusion size to separate the collagen fractions with >3 KDa and <3 KDa (EMD, Millipore Corporation, Billerica, MA, USA). Then, the obtained fractions were freeze-dried. 

The ponderal yields of the freeze-dried HCPs (<3 KDa) named P1 and P2, and those of the HCPs (>3 KDa), named S1 and S2, according to the corresponding starting biomasses F1 and F2, were calculated in comparison with both the total dry weight of the starting fish biomass (F1, F2) and the dry weight of the total protein content, according to the following equations:

(1) $$Yield\left(\%\right)=\frac{weight\;of\;freeze-dried\;HCPs\left(g\right)}{weight\;of\;starting\;dry\;fish\;biomass\;(g)}\times 100$$

(2) $$Yield\left(\%\right)=\frac{weight\;of\;freeze-dried\;HCPs\left(g\right)}{weight\;of\;total\;protein\;content\;(g)}\times 100$$

### 3.7. Sircol Collagen Quantification Assay

The total amount of collagen present in each freeze-dried fraction (P1, P2, S1 and S2) was quantified using the Sircol Collagen Assay Kit, following the manufacturer’s protocol [39]. This method is based on staining with Picrosirius red (F3BA), a strong, linear anionic dye having side-chain groups of sulfonic acid that can bind with the basic amino acid residues in collagens. Briefly, 1 mL of Sircol dye reagent was added to 0.1 mL of the test samples. After 30 min of mild agitation, the solution was centrifugated at 12,000 rpm for 10 min. The obtained pellet was suspended in 0.75 mL of ice-cold acid salt and re-centrifugated at 12,000 rpm for 10 min. A volume of 1 mL of Sodium Hydroxyde 0.5 M (alkali reagent A) was added and the pellet was re-suspended. At the end, the absorbance of the samples was read at 555 nm using a microplate reader (Tecan Spark^®^ 20 M, Tecan, Männedorf, Switzerland). Standard collagen was used to prepare the calibration curve. 

### 3.8. Hydroxyproline Content Assay 

The content of hydroxyproline, a very characteristic amino acid found in collagen, was analyzed in the HCPs fractions (P1, P2, S1 and S2) using a Hydroxyproline Assay Kit (perchlorate-free) from Cell Biolabs (San Diego, CA, USA). Samples were hydrolyzed in 12 N HCl for 3 h at 120 °C.

After evaporation in a heat block for 45 min, the Chloramine T mixture was added and incubated for 30 min at room temperature. Ehrlich’s reagent was added to each sample and incubated for 45 min at 60 °C. Samples were incubated at 4 °C for 5 min and centrifuged at 6000× *g* for 15 min. The samples’ absorbance was read at 555 nm using a microplate reader (Tecan Spark^®^ 20 M, Tecan, Männedorf, Switzerland). A standard curve of hydroxyproline was prepared (100, 50, 25, 12.5, 6.25 µg/mL), following the same procedure as the samples.

### 3.9. Spectrophotometric Analyses 

#### 3.9.1. UV Spectra 

Spectra of the freeze-dried extracted HCPs (P1, P2, S1 and S2), compared to the positive control (CTRL+) solubilized in acetic acid 0.5 M (1 mg/mL), were analyzed using an IMPLEN spectrophotometer (Munich, Germany) in the wavelength range 200–300 nm. Before the measurement of the samples, blank calibration of the UV–Visible spectrophotometer was performed with acetic acid 0.5 M. 

#### 3.9.2. FT-IR Spectroscopy 

Fourier-Transform Infrared Spectroscopy (FT-IR) was applied as a fast, non-destructive, green technique for the qualitative determination of HCPs by using a minimum amount of sample. The infrared spectra of HCPs (P1, P2, S1 and S2) and of the positive control (CTRL+) were obtained using a Perkin Elmer FT-IR spectrophotometer (Inc., Waltham, MA, USA) in the range of 600–4000 cm^–1^. Data were acquired at room temperature with a step resolution of 4 cm^−1^, and for each sample, 8 scans were acquired. 

### 3.10. Cell Viability 

HECV human endothelial cells (Cell Bank and Culture-GMP-IST-Genoa, Genoa, Italy) were cultured at 37 °C in High-Glucose Dulbecco’s modified Eagle’s medium (DMEM) supplemented with 1% L-glutamine and 10% FBS. Cells of 12 × 10^3^ were seeded in each well of a 96-well plate; after one incubation night, cells were treated with different concentrations of collagen extract (50, 25, 16, 12.5, 10, 5, 2.5, 1.6, 1, 0.4, 0.2, 0.13, and 0.1 µg/mL). After 24 h of incubation, the cell viability was quantified using MTT assay [58]; the absorbance of samples was collected at 570 nm using the same plate reader described above. Data were compared to the positive control (CTRL+), a commercial collagen (3 KDa) from ACEF Spa (Fiorenzuola D’arda, PC, Italy) kindly supplied by Ardes s.r.l. (Sarissola, Genova, Italy). All the analyses were performed at least in triplicate. 

### 3.11. T-Scratch Assay

T-scratch assay [59] was performed on HECV human endothelial cells to investigate the migration of the cells. Cells of 150 × 10^3^ were seeded per well in a 12-well plate and incubated overnight. After 24 h, a ‘scratch’ was simulated by scraping the cell monolayer with a sterile 200-pipet tip. Cells were treated with different concentrations of extracts (25, 12.5, 10, 5, 2.5, 1.6, 1, 0.4, 0.2, 0.13, and 0.1 µg/mL) to evaluate their margination capacities after 24 h. For each well, 4 photos were taken using an optical microscope (4× magnification) at time zero and after 24 h. The obtained images were analyzed using ImageJ (1.48 v) software. Data are reported as the percentage of distance comparing the area before and after the treatment. As the positive control, the same commercial collagen reported above was used. All the analyses were performed at least in triplicate. 

## 4. Statistical Analysis

All analyses were carried out in duplicate, unless otherwise stated, and the results were reported as the average value ± the standard error. Data were analyzed using the Excel Data Analysis Tool (Microsoft Corporation, Seattle, WA, USA). Analysis of variance (ANOVA), at a significance level of 0.05, was performed to analyze the mean significant differences among the samples.

## 5. Conclusions

In this study, the whole fish *Mugil cephalus* L., which represented the undersized/“unwanted catches” (sample F1), and the sample F2 made up of unsorted skin, fins, and tails of the same fish, which imitated the by-products of fish processing (e.g., fish fileting), underwent an innovative process for the extraction of HCPs.

The extraction protocol proposed, in order to follow the principles of green chemistry and a circular economy, allowed us to also isolate the intermediate products, namely lipids (about 8–10% of the corresponding biomass weight) and non-collagenous proteins (NCs, 16–23% of the corresponding biomass weight).

The HCPs obtained in the proposed flow-chart, coupling the cavitation process (UAE) to the enzymatic hydrolysis (pepsin), were purified by their molecular weight in the fractions < 3 KDa and > 3 KDa, and we obtained yields of 5–5.2% and 0.04–0.3% (of the corresponding biomass weight), respectively, confirming that the hydrolysis process was quite effective. Moreover, each fraction was characterized (ATR-FTIR spectra, UV spectra, hydroxyproline content assay, and Sircol collagen quantification assay) and then preliminarily biologically tested (cell viability and T-scratch test), since marine collagen is an attractive alternative for different applications (e.g., the cosmetic field).

The use of colorimetric tests, SCA, and hydroxyproline detected the presence of collagen in all the fractions analyzed. In particular, the value of the two fractions, S1 and S2, were higher than the hydrolyzed phases, P1 and P2, due to the presence of more raw material during the extraction process. Cell viability assay on HECV cells determined no significant evidence in terms of cytotoxicity except for the S1 fraction at higher concentrations. Generally, no a negative impact on the cell viability was registered when evaluating all the concentrations tested. Considering the T-scratch test, in general, significant results were shown for all fractions at a 0.13 µg_collagen_/mL concentration, determining that these samples can promote the migration of cells and, consequently, the reimagination of a scratch.

The obtained results suggest that these unsorted materials could be successfully exploited as sources of bioactive compounds, avoiding the expensive and time-consuming process of sorting. In fact, it is important to underline that very often, unwanted catches are not subjected to the filleting procedure, and the separation of their different parts could represent a process too expensive to be both environmentally and economically sustainable.

This study has demonstrated the possibility of exploiting unwanted catches, as an example of undifferentiated biomass, for the extraction of bioactive compounds (i.e., collagen, HCPs) largely used in various sectors such as nutraceuticals and cosmetics. Furthermore, the high yields obtained, both in lipids and in NCs, using the proposed protocol open further interesting possibilities in the exploitation of this biomass.

## Figures and Tables

**Table 1:** Proximate analysis of samples F1, F2 and of the edible portion (fillet).

Sample	Residual Moisture (g/100 g)	Crude Proteins (g/100 g)	Ashes (g/100 g)	Lipids (g/100 g)
F1 (whole fishes)	4.4 ± 0.8	68.7 ± 4.9	16.9 ± 0.5	10.1 ± 0.7
F2 (skin, fins and tails)	4.7 ± 0.3	63.9 ± 2.7	20.6 ± 0.6	8.1 ± 0.4
Edible portion (fillet)	4.0 ± 0.1	75.3 ± 0.9	11.2 ± 0.2	9.0 ± 0.4

Results are expressed as mean value ± standard error.

**Table 2:** Non-collagenous protein (NCPs) yields. Data were expressed as NCPs/total freeze-dried sample weight and as NCPs/total freeze-dried protein weight, respectively.

Sample	NCPs/Total Sample Weight (g/100 g)	NCPs/Total Protein Weight (g/100 g)
F1 (whole fishes)	16.3 ± 0.9	23.7 ± 1.0
F2 (skin, fins and tails)	17.3 ± 0.8	27.1 ± 0.8
Edible portion (fillet)	23.0 ± 0.5	30.5 ± 0.5

Results are expressed as mean value ± standard error.

**Table 3:** HCP extraction ponderal yields obtained from F1 and F2. Data were expressed as HCPs/total freeze-dried sample weight and as HCPs/total freeze-dried protein weight, respectively.

		Ponderal Yield	
Extract Code	Description	HCPs/Total Sample Weight (g/100 g)	HCPs/Total Protein Weight (g/100 g)
P1	<3 KDa (obtained from F1, whole fishes)	5.2 ± 0.3	7.6 ± 0.4
P2	<3 KDa (obtained from F2, skin, fins and tails)	5.0 ± 0.3	7.8 ± 0.3
S1	>3 KDa (obtained from F1, whole fishes)	0.04 ± 0.01	0.6 ± 0.1
S2	>3 KDa (obtained from F2, skin, fins and tails)	0.3 ± 0.1	3.9 ± 0.3

Results are expressed as mean value ± standard error.

**Table 4:** Sircol and hydroxyproline content, expressed as µg/mL, in the obtained extracts (P1, P2, S1 and S2).

Extract Code	SCA (µg/mL)	Hydroxyproline (µg/mL)
CTRL+ P1	60.24 ± 37.30 74.70 ± 0.73	49.32 ± 7.75 3.22 ± 0.45
P2	73.45 ± 3.43	2.41 ± 0.22
S1	141.81 ± 3.83	21.93 ± 7.98
S2	184.02 ± 10.87	33.06 ± 0.68

## Data Availability

Data are contained within the article.
